# Identification of Substance-Exposed Newborns and Neonatal Abstinence Syndrome Using ICD-10-CM — 15 Hospitals, Massachusetts, 2017

**DOI:** 10.15585/mmwr.mm6929a2

**Published:** 2020-07-24

**Authors:** Sonal Goyal, Katherine C. Saunders, Chiara S. Moore, Katherine T. Fillo, Jean Y. Ko, Susan E. Manning, Carrie Shapiro-Mendoza, Munish Gupta, Lisa Romero, Kelsey C. Coy, Kendra B. McDow, Amelia A. Keaton, Jennifer Sinatra, Katarina Jones, Charles Alpren, Wanda D. Barfield, Hafsatou Diop

**Affiliations:** ^1^Division of Reproductive Health, National Center for Chronic Disease Prevention and Health Promotion, CDC; ^2^Epidemic Intelligence Service, CDC; ^3^Massachusetts Department of Public Health; ^4^Beth Israel Deaconess Medical Center, Boston, Massachusetts, ^5^Oak Ridge Institute for Science and Education, Oak Ridge, Tennessee.

Opioid use disorder and neonatal abstinence syndrome (NAS) increased in Massachusetts from 1999 to 2013 (*1*,*2*). In response, in 2016, the state passed a law requiring birth hospitals to report the number of newborns who were exposed to controlled substances to the Massachusetts Department of Public Health (MDPH)* by mandating monthly reporting of *International Classification of Diseases, Tenth Revision, Clinical Modification* (ICD-10-CM) diagnostic codes related to maternal dependence on opioids (F11.20) or benzodiazepines (F13.20) and to newborns affected by maternal use of drugs of addiction (P04.49) or experiencing withdrawal symptoms from maternal drugs of addiction (P96.1) separately.^†^ MDPH uses these same codes for monthly, real-time crude estimates of NAS and uses P96.1 alone for official NAS state reporting.^§^ MDPH requested CDC’s assistance in evaluating the sensitivity, specificity, positive predictive value (PPV), and negative predictive value (NPV) of either maternal or newborn codes to identify substance-exposed newborns, and of newborn exposure codes (both exposure [P04.49] or withdrawal [P96.1]) and the newborn code for withdrawal alone (P96.1) to identify infants with NAS cases related to three exposure scenarios: 1) opioids, 2) opioids or benzodiazepines, and 3) any controlled substance. Confirmed diagnoses of substance exposure and NAS abstracted from linked clinical records for 1,123 infants born in 2017 and their birth mothers were considered the diagnostic standard and were compared against hospital-reported ICD-10-CM codes. For identifying substance-exposed newborns across the three exposure scenarios, the newborn exposure codes had higher sensitivity (range = 31%–61%) than did maternal drug dependence codes (range = 16%–41%), but both sets of codes had high PPV (≥74%). For identifying NAS, for all exposure scenarios, the sensitivity for either newborn code (P04.49 or P96.1) was ≥92% and the PPV was ≥64%; for P96.1 alone the sensitivity was ≥79% and the PPV was ≥92% for all scenarios. Whereas ICD-10-CM codes are effective for NAS surveillance in Massachusetts, they should be applied cautiously for substance-exposed newborn surveillance. Surveillance for substance-exposed newborns using ICD-10-CM codes might be improved by increasing the use of validated substance-use screening tools and standardized facility protocols and improving communication between patients and maternal health and infant health care providers.

The evaluation examined the validity of using ICD-10-CM codes to estimate the prevalence of substance-exposed newborns and NAS in Massachusetts among 15 hospitals identified by MDPH from among 41 Massachusetts birthing hospitals.^¶^ During the planning and development of protocols and methods, the most recent year for which data were complete was 2017; the evaluation was conducted in the first quarter of 2019. All 33,431 live-born infants in 2017 from the identified hospitals were linked to their mother’s record and were categorized into three mutually exclusive groups: 1) infants or their mothers assigned specific maternal or newborn ICD-10-CM codes (related to maternal drug dependence or newborn exposure or withdrawal) as reported to MDPH by hospitals, regardless of risk factors**; 2) mother-infant pairs with risk factors associated with an increased likelihood of substance use during pregnancy (*3*–*5*) but without the specified ICD-10-CM codes^††^; and 3) infants not in the first two groups. A total of 1,129 infant-mother pairs were selected using stratified sampling from within those three groups at each hospital; infants in the first group (those with specific ICD-10-CM codes assigned) were oversampled to increase the probability of identifying false positives.^§§^

The validity (sensitivity, specificity, PPV, and NPV) of the following ICD-10-CM code combinations to assess substance-exposed newborns were calculated: 1) those related to maternal dependence on opioids (F11.20) or benzodiazepines (F13.20) and 2) those related to newborns affected by maternal drug exposure (P04.49) or experiencing withdrawal from drug exposure (P96.1). To identify NAS, the validity of codes P04.49 or P96.1 and P96.1 alone were assessed. Analyses conducted using P96.1 alone included infants from 12 of the 15 hospitals (69.5% of the weighted sample) that reported individual ICD-10-CM codes to MDPH. Substance-exposed newborns and NAS that were confirmed using abstracted clinical record data served as the diagnostic standard. Identification of substance-exposed newborns was confirmed using either a documented history of maternal substance use during pregnancy or laboratory confirmation^¶¶^ of maternal drug use or fetal exposure to selected controlled substances within the 30 days preceding delivery.*** Three substance exposure scenarios were assessed: 1) exposure to opioids, 2) exposure to opioids or benzodiazepines, and 3) exposure to opioids, benzodiazepines, barbiturates, amphetamines, cocaine, hallucinogens, or marijuana (i.e., any controlled substance). Infants were confirmed as having NAS if 1) newborn substance exposure was confirmed and a Finnegan or modified Finnegan score (a system used to quantify and diagnose NAS) was ≥8 (*6*) or 2) if diagnosis of NAS was officially documented in the infant’s medical record.^†††^ The final sample was weighted to represent the total number of births from each of the three groups at each selected hospital. Multiparous births were adjusted to account for nonindependence between related infants. Prevalence of characteristics of the total sample and both substance-exposed and nonexposed newborns and 95% confidence intervals were calculated. Analyses were conducted using SAS (version 9.4; SAS Institute).

Records for 1,123 mother-infant pairs were abstracted; six infants were excluded because there was insufficient information in the clinical chart to confirm that the birth mother and infant were linked correctly. The data included information from four complete sets of twins and 33 other infants who constituted one member of multiparous births. Most infants were born at term (92%) and weighed >2,500 g at birth (91%) (Table 1). Approximately one third of mothers were aged 30–34 years (36%) and nearly one half (47%) were non-Hispanic white. Across all exposure scenarios for substance-exposed newborns, the sensitivity of newborn exposure codes (P04.49 or P96.1) was ≥14 percentage higher (range = 31%–61%) than were maternal drug dependence codes (F11.20 or F13.20) (range = 16%–41%) (Table 2). Sensitivity for identifying substance-exposed newborns was highest in the exposure to opioids only scenario when evaluating maternal and newborn codes. The PPV for both the newborn exposure codes and maternal drug dependence codes was high for substance-exposed newborns (≥74%) across all exposure scenarios. Evaluating NAS for all exposure scenarios, the sensitivity of P04.49 (exposure) or P96.1 (withdrawal) (≥92%) was higher than that for P96.1 alone (≥79%). The PPV for P04.49 or P96.1 was lower (64%–65%) than that of P96.1 alone (≥92%). All ICD-10-CM code combinations had high specificity and NPV (≥94%) for all exposure scenarios for substance-exposed newborns and NAS.

**TABLE 1:** Weighted characteristics of mother-infant pairs included in the study population as a percentage of the total sample, substance-exposed newborns, and non–substance-exposed newborns* — 15 Massachusetts hospitals, 2017

Characteristic	Newborns % (95% CI)		
	Total sample, unweighted N = 1,123	Substance-exposed, unweighted n = 470	Non–substance-exposed, unweighted n = 653
**Maternal age group (yrs)**			
<20	3.5 (2.0–5.0)	6.0 (0.0–13.1)	3.3 (1.8–4.9)
20–24	10.9 (8.4–13.4)	24.5 (12.8–36.3)	9.9 (7.5–12.4)
25–29	25.8 (22.5–29.2)	23.9 (14.6–33.3)	26.0 (22.4–29.5)
30–34	36.3 (32.6–40.0)	30.4 (18.5–42.3)	36.7 (32.8–40.6)
≥35	23.5 (20.0–26.9)	15.1 (4.2–26.0)	24.1 (20.4–27.7)
**Maternal race and ethnicity^†^**			
White, non-Hispanic	47.1 (43.1–51.0)	54.6 (41.4–67.7)	46.5 (42.4–50.6)
Hispanic	19.4 (16.2–22.5)	21.1 (9.6–32.6)	19.3 (16.0–22.5)
Black, non-Hispanic	7.5 (5.6–9.4)	6.4 (0.0–13.7)	7.6 (5.6–9.6)
Asian, non-Hispanic	7.1 (5.1–9.0)	2.3 (0.0–6.6)	7.4 (5.4–9.5)
Other	2.7 (1.4–4.0)	1.0 (0.3–1.6)	2.8 (1.4–4.2)
Unknown/Missing	16.3 (13.4–19.1)	14.6 (5.4–23.8)	16.4 (13.4–19.4)
**Infant sex**			
Male	50.7 (46.8–54.7)	57.8 (45.0–70.6)	50.2 (46.1–54.4)
**Infant gestational age at birth (wks)**			
<34	2.7 (1.4–3.9)	3.5 (0.5–6.6)	2.6 (1.3–3.9)
34–36	5.4 (3.7–7.0)	9.6 (2.7–16.6)	5.1 (3.4–6.8)
≥37 (term)	92.0 (89.9–94.0)	86.8 (79.3–94.4)	92.3 (90.2–94.4)
**Infant birthweight (grams)**			
500–1,499	1.4 (0.5–2.3)	1.0 (0.4–1.7)	1.4 (0.4–2.3)
1,500–2,499	7.2 (5.2–9.2)	12.6 (5.2–20.0)	6.8 (4.8–8.8)
≥2,500	91.4 (89.3–93.6)	86.4 (78.9–93.8)	91.8 (89.6–94.0)
**Multiple live births**			
Singleton	97.3 (96.1–98.5)	98.4 (97.6–99.3)	97.2 (95.9–98.5)
Multiples	2.7 (1.5–3.9)	1.6 (0.7–2.4)	2.8 (1.5–4.1)
**Highest Finnegan NAS score^§,¶^**			
Mean (range)	—	9.5 (2–28)	NR**
**Length of hospital stay (days)**			
Mean (range)	4.2 (0–155)	10.2 (0–155)	3.8 (0–142)
Median	1.8	2.3	1.8

**Abbreviations:** CI = confidence interval; NAS = neonatal abstinence syndrome; NR = not reported.

* Missing included if >5%.

^†^ If more than two races were chosen, the maternal race and ethnicity were identified as other; if Hispanic ethnicity was unknown or if race was unknown, the maternal race and ethnicity was labeled as unknown/missing.

^§^ A scored assessment of the most common signs of neonatal abstinence syndrome.

^¶^ Only 2.1% of the weighted total sample had reported Finnegan scores.

** Not reported because number was <5.

**TABLE 2:** Sensitivity, specificity, positive predictive value, and negative predictive value of reported *International Classification of Diseases, Tenth Edition, Clinical Modification* (ICD-10-CM) codes compared with confirmed cases of substance-exposed newborns and infants with neonatal abstinence syndrome caused by in utero exposures to various substance groups, by type of controlled substance — 15 Massachusetts hospitals, 2017

ICD-10-CM code	Validation measure	Substance-exposed newborns and infants with NAS, % (95% CI)*		
		Opioids	Opioids or benzodiazepines	Opioids, benzodiazepines, barbiturates, marijuana, amphetamines, cocaine, or hallucinogens
**Substance-exposed newborns**				
F11.20 or F13.20^†^	Sensitivity	41.4 (28.1–54.8)	30.9 (20.7–41.0)	16.3 (11.6–21.0)
	Specificity	100.0 (100.0–100.0)	100.0 (100.0–100.0)	100.0 (100.0–100.0)
	PPV	98.6 (96.9–100.0)	98.9 (97.3–100.0)	98.9 (97.3–100.0)
	NPV	98.5 (97.6–99.3)	97.5 (96.4–98.6)	94.4 (92.7–96.1)
P04.49 or P96.1^†^	Sensitivity	60.5 (41.7–79.3)	45.7 (31.2–60.2)	30.9 (22.5–39.2)
	Specificity	99.4 (99.3–99.5)	99.5 (99.3–99.6)	99.9 (99.9–100.0)
	PPV	73.9 (69.6–78.3)	75.2 (70.9–79.5)	96.0 (94.1–98.0)
	NPV	98.9 (98.1–99.8)	98.1 (96.9–99.2)	95.3 (93.6–97.0)
**Infants with neonatal abstinence syndrome**				
P04.49 or P96.1^†^	Sensitivity	92.1 (88.8–95.5)	92.2 (88.9–95.5)	92.3 (89.0–95.5)
	Specificity	99.2 (99.1–99.4)	99.2 (99.1–99.4)	99.2 (99.1–99.4)
	PPV	63.9 (59.1–68.6)	64.3 (59.6–69.1)	65.0 (60.3–69.7)
	NPV	99.9 (99.8–99.9)	99.9 (99.8–99.9)	99.9 (99.8–99.9)
P96.1^†,§^	Sensitivity	80.2 (74.3–86.1)	79.8 (73.9–85.7)	79.4 (73.5–85.3)
	Specificity	99.9 (99.8–99.9)	99.9 (99.8–99.9)	99.9 (99.8–99.9)
	PPV	91.7 (87.9–95.5)	92.0 (88.3–95.7)	92.3 (88.6–96.0)
	NPV	99.7 (99.6–99.8)	99.7 (99.6–99.8)	99.7 (99.6–99.8)

**Abbreviations:** CI = confidence interval; NAS = neonatal abstinence syndrome; NPV = negative predictive value; PPV = positive predictive value.

* Percentages use weighted data.

^†^ F11.20: opioid dependence, uncomplicated; F13.20: sedative, hypnotic, or anxiolytic dependence, uncomplicated; P04.49: newborn affected by maternal use of other drugs of addiction; and P96.1: neonatal withdrawal symptoms from maternal use of drugs of addiction.

^§^ Weighted data from 12 of 15 selected hospitals that reported individual ICD-10-CM diagnostic codes (representing 69.5% of total weighted sample).

## Discussion

ICD-10-CM codes can be used to monitor the prevalence of several conditions, including substance-exposed newborns and NAS. Evaluating the sensitivity and PPV of these codes can inform how well they identify actual cases. The MDPH surveillance system reports selected maternal drug dependence codes and newborn exposure codes for monitoring substance-exposed newborns separately, and this analysis found the newborn exposure codes to have higher (although still low to moderate) sensitivity than do maternal drug dependence codes within each substance exposure scenario; specificity for both types of codes was consistently high.

These data demonstrate opportunities for improvement in identifying substance-exposed newborns. Implementing universal maternal screening protocols with validated screening tools to help identify maternal drug use, executing standardized facility protocols around screening and assessing newborns for substance exposure, and improving communication between patients and maternal and infant health care providers might lead to improvement in identifying substance-exposed newborns (*7*), which could lead to more accurate assignment of ICD-10-CM codes.^§§§^ Sensitivity of ICD-10-CM codes was consistently highest when evaluating substance-exposed newborns with opioid exposure only, even though the ICD-10-CM codes aren’t specific to only opioids. Efforts by providers and medical coders to assign appropriate ICD-10-CM codes for nonopioid exposure could increase ICD-10-CM code sensitivity for nonopioid substances.

State surveillance definitions of NAS vary widely across the United States (*8*). Most states use only P96.1 (withdrawal) for identifying NAS because P04.49 is primarily used to identify substance-exposed newborns (*8*). A recent Tennessee study using ICD-10-CM codes found a high PPV (98%) for P96.1 to identify NAS caused by opioids (*9*), consistent with the findings (92%) of this evaluation. In Massachusetts, codes for exposure (P04.49) or withdrawal (P96.1) might yield the most sensitive estimates for identifying infants with NAS, but P96.1 alone better identifies infants who indeed have NAS because of its higher PPV; however, PPV varies by population prevalence.

Using exposure (P04.49) in addition to withdrawal (P96.1) codes might be more sensitive for identifying NAS than using P96.1 alone because P04.49 might identify newborns exhibiting signs of NAS who have not received a diagnosis of withdrawal (P96.1) by providers. Because these codes provide different information, they should be selected based on the surveillance purpose. In Massachusetts, many hospitals have programs to support mothers with substance use disorder and infants with a diagnosis of NAS (*10*). Identifying infants with NAS in real time is important for linking families to these programs and evaluating their impact; therefore, a more sensitive NAS surveillance system could help ensure that all families that might potentially benefit from the programs are linked to them. However, a code with higher PPV will better identify newborns who genuinely have NAS and might be more accurate for tracking state estimates. In contrast to findings assessing ICD-10-CM codes to identify substance-exposed newborns, the sensitivity of ICD-10-CM codes for identifying NAS was similar across all three exposure scenarios. Although NAS is a more general term for neonatal withdrawal that can include nonopioid exposures (e.g., benzodiazepines), evidence suggests that the recent increases in NAS are primarily from in utero exposure to opioids, either alone or in combination with other substances; in this analysis, nearly all (98%) of the newborns with NAS were exposed to opioids.

The findings in this report are subject to at least four limitations. First, because of stigma and legal implications, disclosure of maternal use of controlled substances and NAS diagnosis might be underreported by patients and clinical providers, resulting in reporting bias and leading to underreporting in the clinical records used as the standard.^¶¶¶^ However, multiple sources of information were used to determine controlled substance use and exposure, including any notation of Finnegan score and available laboratory results (urine, meconium, and blood) to increase sensitivity of ascertainment. Second, the current ICD-10-CM used to monitor substance-exposed newborns might affect coding because the maternal dependence codes are specific to opioids and benzodiazepines only, but the newborn substance exposure codes do not specify distinct substances. Third, accuracy and consistency of coding might vary by facility. Finally, results are only generalizable to the 15 selected Massachusetts hospitals. Data were limited by the exclusion of small birthing facilities, and, because some facilities did not report P96.1 separately from P04.49, three of 15 hospitals were excluded when evaluating ICD-10-CM code P96.1 alone, resulting in a total of 12 for analysis.

This evaluation contributes to understanding the use of ICD-10-CM codes for assessing the public health prevalence of substance-exposed newborns and NAS in Massachusetts. Considering the exposures of interest and the purpose of surveillance, public health organizations, including MDPH, might effectively conduct surveillance for NAS using ICD-10-CM codes. Surveillance for substance-exposed newborns using ICD-10-CM codes in Massachusetts should be undertaken with caution at this time but might be improved by increasing the use of validated substance-use screening tools and standardized facility protocols and improving communication between patients and maternal health and infant health care providers.

**Summary**

**What is already known about the topic?**

Massachusetts uses independent combinations of *International Classification of Diseases, Tenth Revision, Clinical Modification* (ICD-10-CM) diagnostic codes to surveil substance-exposed newborns and neonatal abstinence syndrome (NAS), but the ability of these codes to identify substance-exposed newborns and NAS is unknown.

**What is added by this report?**

Whereas ICD-10-CM codes performed relatively well for surveillance of NAS in the sample for this study (sensitivity range = 79%–92%, positive predictive value range = 64%–92%), surveillance for substance-exposed newborns using ICD-10-CM codes missed more cases (sensitivity range = 16%–61%).

**What are the implications for public health practice?**

In Massachusetts, ICD-10-CM codes are effective for NAS surveillance but should be applied cautiously for surveillance of substance-exposed newborns.

## Footnotes

* https://malegislature.gov/Laws/SessionLaws/Acts/2016/Chapter52.

† The following are definitions of the ICD-10-CM diagnostic codes required for reporting by Massachusetts: F11.20 = opioid dependence, uncomplicated; F13.20 = sedative, hypnotic, or anxiolytic dependence, uncomplicated; P04.49 = newborn affected by maternal use of other drugs of addiction [besides unspecified drugs of addiction, cocaine and hallucinogens]; and P96.1 = neonatal withdrawal symptoms from maternal use of drugs of addiction.

§ Massachusetts has two statewide NAS surveillance systems. One uses ICD-10-CM codes P96.1 or P04.49 to provide rapid, crude estimates of NAS for monthly facility-based NAS reporting. Because NAS is more likely to be diagnosed in cases that require pharmacologic intervention, MDPH includes P04.49 in addition to P96.1. The second system uses ICD-10-CM code P96.1 (and its equivalent, *International Classification of Diseases, Ninth Revision, Clinical Modification* (ICD-9-CM) code 779.5) to identify NAS cases by linking hospital discharge data to birth certificate data in the Pregnancy to Early Life Longitudinal (PELL) data system. These codes are automatically recorded in the PELL data system, which provides cleaned, reliable data that include quality of care indicators and covariates to assess health disparities. https://www.mass.gov/guides/neonatal-abstinence-syndrome-dashboard#-explore-the-nas-data-dashboard-.

¶ Five of 46 birthing hospitals in Massachusetts were excluded because they had <300 births. The remaining 41 were stratified with a quasirandom design by the six Massachusetts Executive Office of Health and Human Services Regions (West, Central, Northeast, Metro, Southeast, and Metro-North), and further stratified by prevalence of substance-exposed newborns below or at or above the mean (three per 100 live births) as reported in 2017 in Massachusetts. Fifteen hospitals were randomly selected from these 12 strata, based on the total number of hospitals in each stratum. One hospital was substituted to ensure representation by teaching status and hospital system.

** The specific diagnostic codes included infants who were assigned P96.1 or P04.49 during their birth hospitalization or infants whose birth mothers were assigned F11.20 or F13.20 during pregnancy based on hospital reporting to MDPH.

†† Risk factors were based upon previously published analyses and included birth mother’s age <34 years at birth event, birth mother’s insurance through Medicaid, or infant length of stay ≥5 days.

§§ The aim was to oversample from group 1 and from group 2 to increase the likelihood of identifying false-positives and false-negatives, respectively. The initial goal was to capture a final unweighted sample comprising 40% of infant-mother pairs in group 1, 50% in group 2, and 10% in group 3. The final unweighted sample (the 1,123 records abstracted) comprised 462 (41.1%), 549 (48.9%), and 112 (10.0%) infants-mother pairs in groups 1, 2 and 3, respectively. Among the 33,431 infants at the 15 hospitals, 59.5%, 1.9%, and 2.1% of infants from groups 1, 2, and 3, respectively, were included in the sample. Because the sample had approximately 2% of all infants in groups 2 and 3, but 59.5% of the infants in group 1, infant-mother pairs in group 1 only were oversampled.

¶¶ Maternal urine toxicology laboratory testing results 30 days before through 2 days after delivery were assessed to ascertain substance use 30 days before and up to delivery; metabolites of the substances of interest remain detectable in urine 2 days after their use. https://ncsacw.samhsa.gov/files/IA_Drug_Testing_Bench_Card_508.pdf.

*** Recorded substance use was not limited to illicit use. Drugs used for delivery were not included as substance exposures.

††† In the sample, only Finnegan or modified Finnegan scores were identified in clinical charts. Two infants were assessed using Eat, Sleep, Console (https://www.nature.com/articles/s41372-020-0733-y), but results were not documented in the clinical record.

§§§ https://www.samhsa.gov/sites/default/files/topics/specific_populations/protecting-our-infants-act-report-congress-2017.pdf.

¶¶¶ https://www.guttmacher.org/state-policy/explore/substance-use-during-pregnancy.
